# Influence of adherence with guideline-driven recommendations on survival in women operated for breast cancer: Real-life evidence from Italy

**DOI:** 10.1016/j.breast.2020.06.010

**Published:** 2020-07-01

**Authors:** Giovanni Corrao, Federico Rea, Enza Di Felice, Mirko Di Martino, Marina Davoli, Luca Merlino, Flavia Carle, Rossana De Palma, “Monitoring and assessing diagnostic-therapeutic paths (MAP)” of the Italian Ministry of Health

**Affiliations:** a National Centre for Healthcare Research and Pharmacoepidemiology, Department of Statistics and Quantitative Methods, University of Milano-Bicocca, Milan, Italy; b Unit of Biostatistics, Epidemiology and Public Health, Department of Statistics and Quantitative Methods, University of Milano-Bicocca, Milan, Italy; c Authority for Healthcare and Welfare, Emilia Romagna Regional Health Service, Bologna, Italy; d Department of Epidemiology, Lazio Regional Health Service, Roma, Italy; e Epidemiologic Observatory, Lombardy Region Welfare Department, Milan, Italy; f Center of Epidemiology and Biostatistics, Polytechnic University of Marche, Ancona, Italy

**Keywords:** Breast cancer, Guideline-driven recommendations, Survival, Healthcare utilization database, Care pathways

## Abstract

**Background:**

A set of indicators to assess the quality of care for women operated for breast cancer was developed by an expert working group of the Italian Health Ministry in order to compare the Italian regions. A study to validate these indicators through their relationship with survival was carried out.

**Methods:**

The 16,753 women who were residents in three Italian regions (Lombardy, Emilia-Romagna and Lazio) and hospitalized for breast cancer surgery during 2011 entered the cohort and were followed until 2016. Adherence to selected recommendations (i.e., surgery timeliness, medical therapy timeliness, appropriateness of complementary radiotherapy and mammographic follow-up) was assessed. Multivariable proportional hazards models were fitted to estimate hazard ratios for the association between adherence with recommendations and the risk of all-cause mortality.

**Results:**

Adherence to recommendations was 53% for medical therapy timeliness, 73% for appropriateness of mammographic follow-up, 74% for surgery timeliness and 82% for appropriateness of complementary radiotherapy. Risk reductions of 26%, 62% and 56% were observed for adherence to recommendations on medical therapy timeliness, appropriateness of complementary radiotherapy and mammographic follow-up, respectively. There was no evidence that mortality was affected by surgery timeliness.

**Conclusions:**

Clinical benefits are expected from improvements in adherence to the considered recommendations. Close control of women operated for breast cancer through medical care timeliness and appropriateness of radiotherapy and mammographic monitoring must be considered the cornerstone of national guidance, national audits, and quality improvement incentive schemes.

## Highlights

• 4 indicators to assess the quality of care in operated breast cancer were developed.
• High heterogeneity across regions in breast cancer-related care was observed.
• Almost all the indicators are associated with improved overall survival.
• Benefits are expected from improving adherence with the considered recommendations.

## 1 Introduction

Quality of healthcare refers to “… the degree to which healthcare services … increase the likelihood of desired health outcomes …” [1]. Several sources provide data for measuring quality of healthcare, but in the absence of a coordinated national quality measurement and reporting system, these sources are likely to be unsuitable for a national overview of the quality of available healthcare choices [2].

Breast cancer is the most common cancer in women worldwide. In 2012, nearly 1.7 million women were diagnosed with breast cancer and 6.2 million women had received a prior breast cancer diagnosis [3]. Resolutions of the European Parliament on June 2003 and October 2006 call on the European Union (EU) member states to make the fight against breast cancer a health policy priority and to develop and implement effective strategies for improved health care encompassing screening, diagnosis and treatment throughout Europe [4]. Accordingly, guidelines for breast cancer-related follow-up care were developed by several scientific societies (e.g., the American Society for Clinical Oncology (ASCO) [5]). In 2014, the European Society for Breast Cancer Specialists (EUSOMA) updated a set of quality indicators to be adopted by breast centres to allow quality assurance and to establish an agreed minimum standard of care [6]. In general, however, measuring adherence to such guidelines, and the resulting recommendations, involves development of indicators that are so detailed as to be largely unsuitable for comparing quality of care across large populations (e.g., those benefiting from insurance companies or a National Health Service, NHS).

A system for assessing integrated care pathways for specific clinical conditions is being developed by an Italian group of experts of the Italian Health Ministry, the so-called Monitoring and Assessing diagnostic-therapeutic Paths (MAP) working group [7]. As healthcare management in Italy is provided by the Regions, in developing the system of indicators, the MAP working group paid particular attention to what can actually be measured by, and compared among, the Italian regions. Another constraint considered by the working group was that, in the absence of robust evidence based on clinical trials, indicators do not necessarily measure quality of care, so that validating studies estimating their effectiveness in improving the desired health outcomes became essential.

The set of process indicators for measuring the quality of care in operated breast cancer took inspiration from indicators of scientific societies (in particular those of the Italian Association of Medical Oncology (AIOM) [8]). A small number of measurable and comparable indicators were developed. In addition, given that a better process profile, as measured by these indicators, does not necessarily translate into better outcomes, a study for validating the set of indicators through their relationship with measurable clinical outcomes was designed.

In view of these preliminary remarks, this paper reports the methods and findings of validating indicators, and discusses their implications, in order to compare quality of care for women operated for breast cancer across the Italian Regions, from a national perspective.

## 2 Materials and methods

### 2.1 Setting

All Italian citizens have equal access to health care services as part of the NHS. An automated system of healthcare utilization (HCU) databases allows each Italian region to manage the NHS at local level. HCU data include a variety of information on residents who receive NHS assistance, diagnosis on discharge from public or private hospitals, outpatient drug prescriptions, specialist visits and diagnostic exams reimbursable by the NHS, and co-payment exceptions for diagnosed chronic diseases, including cancer.

As a unique identification code is systematically used for all databases within each region, records can be linked to enable searches on the complete care pathways of NHS beneficiaries. In order to preserve privacy, identification codes are automatically converted into anonymous codes, the inverse process being allowed only to the Regional Health Authority on request from judicial authorities.

### 2.2 Harmonization and data processing

This study is based on computerized HCU databases from three Italian Regions (Lombardy, Emilia-Romagna and Lazio). Approximately 20 million Italian NHS beneficiaries were registered in the corresponding databases, accounting for nearly one-third of the Italian population.

Although the databases did not differ substantially across the Regions, an inter-region data harmonization was performed, thus allowing data extraction processes to target the same semantic concepts (e.g., information was uniformly encoded by using the same names, values and formats). Anonymized data were extracted and processed locally using a common Statistical Analysis System (SAS) program developed by two members of our group (FR and EDF) in accordance with the protocol previously approved by the MAP working group.

The specific diagnostic and therapeutic codes used for the current study are provided in Supplementary material (Table S1).

### 2.3 Cohort selection and follow-up

NHS beneficiaries of the female gender who in the index year (i.e., in 2011) were 18 years or older and resident in one of the three participating Italian Regions formed the target population. Among these, women who were hospitalized for breast cancer surgery were identified, and the first hospital admission during 2011 was defined as the index hospitalization. In order to ensure sufficient time back for patient characterization through their previous contacts with the NHS, subjects were excluded if they were recorded as beneficiaries of the regional NHS after the year 2007. The exclusion was extended to three other categories of women, i.e., those who had received a diagnosis of (i) breast cancer more than 6 months before index hospitalization, (ii) other forms of cancer during the time-span ranging from three years before and six months after index hospitalization, and (iii) malignant neoplasm within 3 years of index hospitalization.

The remaining patients were included in the final cohort whose members accumulated person-years of follow-up from the date of discharge of the index hospitalization (index discharge) until the occurrence of one of the following events, whichever came first: the study outcome (death), emigration, or end-point of follow-up, i.e., December 31, 2016.

### 2.4 Adherence with recommendations

Recommendations covered surgery timeliness, medical therapy timeliness, appropriateness of complementary radiotherapy and mammographic follow-up.

Surgery timeliness was evaluated in the restricted cohort of women who (i) underwent mammography in the six months before index hospitalization, and (ii) did not receive neoadjuvant therapy in the same period. These women were classified as adherent with the surgery timeliness recommendation if they underwent mammography at least once in the 2 months before index hospitalization, otherwise they were classified as non-adherent.

Medical therapy timeliness was evaluated in the restricted cohort of women who (i) did not receive neoadjuvant therapy in the six months before index hospitalization, (ii) did not receive exclusive radiotherapy within six months after index discharge, (iii) were not re-hospitalized for breast surgery within four months after index discharge, and (iv) accumulated at least 45 days of follow-up. These women were classified as adherent with the medical therapy timeliness recommendation if they started chemotherapy within 45 days after index discharge, otherwise they were classified as non-adherent.

Appropriateness of complementary radiotherapy was evaluated in the restricted cohort of women who (i) had a diagnosis of invasive breast cancer, (ii) underwent breast-conserving surgery during the index hospital stay, (iii) underwent chemotherapy within six months after index discharge, and (iv) accumulated at least twelve months of follow-up. These women were classified as adherent with the appropriateness of complementary radiotherapy recommendation if they started radiotherapy within twelve months from index discharge, otherwise they were classified as non-adherent.

Appropriateness of mammographic follow-up was evaluated in the restricted cohort of women who accumulated at least eighteen months of follow-up. These women were classified as adherent with the appropriateness of mammographic follow-up recommendation if they underwent mammography at least once within eighteen months from index discharge, otherwise they were classified as non-adherent.

### 2.5 Covariates

The baseline characteristics of cohort members included age, type of breast cancer (invasive cancer or carcinoma in situ) and type of surgery (breast-conserving surgery or mastectomy). In addition, data on mammography and neoadjuvant therapy in the six months before index hospitalization were recorded. Finally, the so-called Multisource Comorbidity Score (MCS), a simple score recently developed and validated in Italy [9], was used to assess the clinical profile of each cohort member. In this study, the weights of the conditions that contribute to the score were re-estimated by considering the cohort of cancer patients [10], rather than the general population as in the original version of the MCS (Supplementary material, Table S2).

### 2.6 Data analysis

We used the following two-stage procedure for generating pooled meta-analytic estimates of adherence-outcome association.

In the first stage, four Cox proportional hazard regression models, one for each process indicator, were fitted within each participant region to estimate separately the hazard ratio (HR), and its 95% confidence interval (CI), of all-cause mortality in relation to adherence with recommendations. These models were fitted among the specific sub-cohorts in which the process indicators were defined and assessed. Adjustments were made for the above-listed covariates.

In order to estimate the summarized adherence-outcome association, in the second stage a random-effects meta-analysis [11] was performed to combine the HRs obtained from the Regions considered [12]. Between-region heterogeneity was tested with Cochran’s Q test and measured with the I^2^ statistics that is the proportion of between-region variability due to heterogeneity [13].

### 2.7 Sensitivity analyses

To assess the robustness of the main findings, three sensitivity analyses were performed, restricted to women from Lombardy, the largest of the Regions participating in the study.

First, because the thresholds adopted to define adherence with recommendations were arbitrary, the main criteria were modified as follows: (i) surgery timeliness: undergoing at least a mammography in the 1 or 3 months before index hospitalization, (ii) medical therapy timeliness: starting chemotherapy within 30 or 60 days of the date of index discharge, (iii) appropriateness of complementary radiotherapy: starting radiotherapy within 9 or 15 months of the date of index discharge, and (iv) appropriateness of mammographic follow-up within 12 or 24 months after index hospitalization.

Second, breast cancer mortality was considered as the outcome. Proportional hazard regression models proposed by Fine and Gray [14] were fitted to estimate the HR for the association between adherence with recommendations and breast cancer mortality accounting for the competing risk of death from any other cause.

Third, as adherence was likely affected by clinical profile and other relevant characteristics, and because the information available from healthcare databases like ours was limited, a 1:1 high-dimensional propensity score (HDPS) matching design was adopted. Exposure propensity scores were derived from the HDPS algorithm, an automated technique that identifies and prioritizes covariates that may serve as proxies for unmeasured confounders in large electronic healthcare databases [15]. In brief, the predicted probability of adherence with recommendations was estimated for each cohort member through a logistic regression model, whose covariates were the above-mentioned baseline data, plus all the possible causes of hospital discharge experienced by, and drugs prescribed to, cohort members in the 2-year period prior to index admission. The 200 most predictive covariates were selected. Groups were 1:1 matched on their propensity score using a nearest neighbour matching algorithm without replacement [16].

All analyses were performed using the SAS Software (version 9.4; SAS Institute, Cary, NC). For all hypotheses tested, 2-tailed p-values less than 0.05 were considered significant.

## 3 Results

### 3.1 Patients

**Fig. 1:**
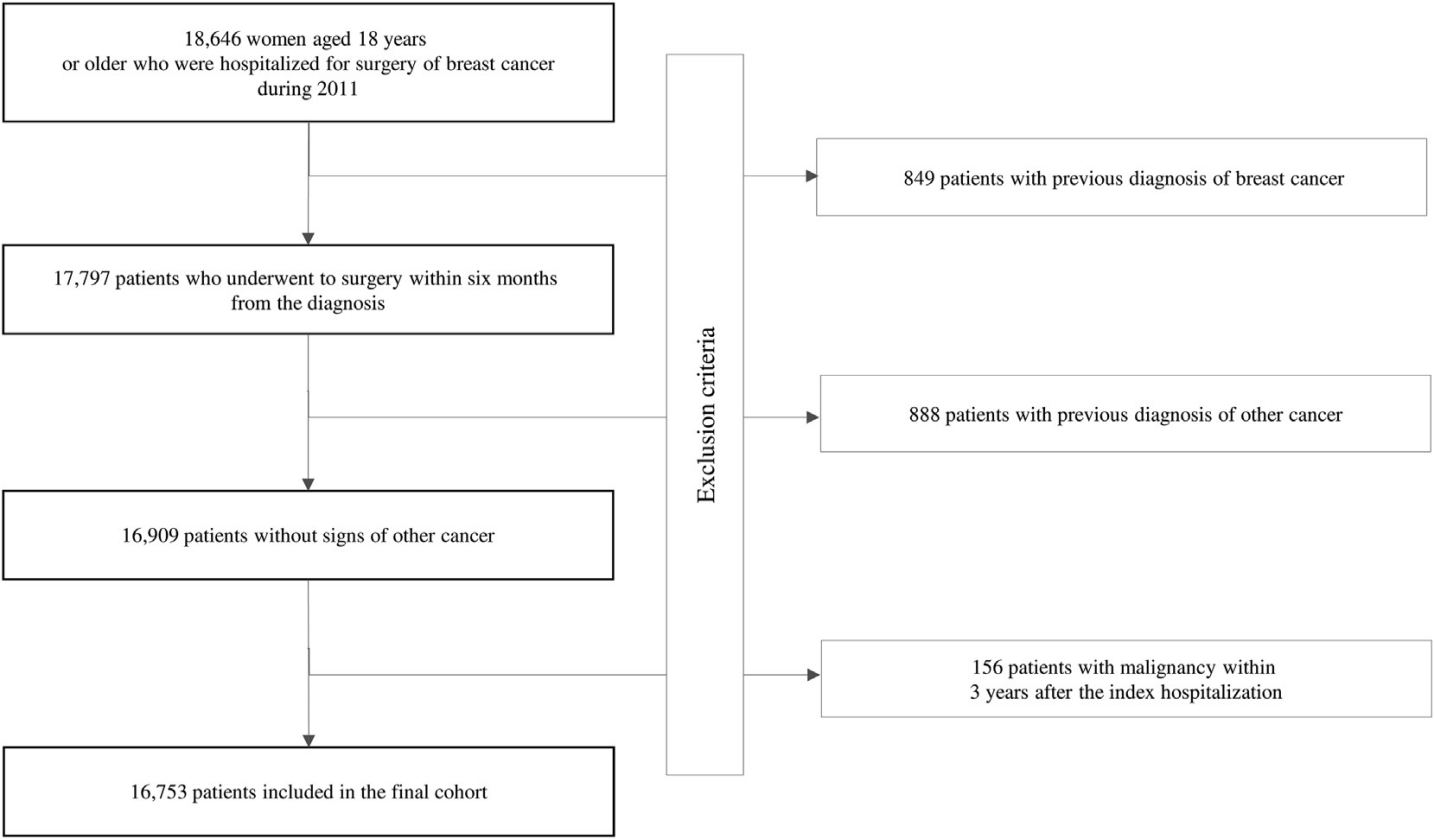
Flow-chart of inclusion and exclusion criteria. Italy, 2011–2016.

Among the almost 19,000 eligible women, 16,753 met the inclusion criteria and formed the study cohort (Fig. 1). The patients included in the final cohort accumulated 78,304 person-years (PYs) (on average 4.7 years per woman) and generated 1906 deaths, with an all-cause mortality rate of 24.3 every 1000 PYs.

The baseline characteristics of the cohort members from each participant region, as well as of the aggregate cohort, are shown in Table 1. Almost half of the women were aged 65 years or older, nine out of ten patients had a diagnosis of invasive cancer and almost three quarters underwent breast-conserving surgery. Finally, most women had none or just a few comorbidities (0≤MCS<2). There was evidence that all baseline characteristics differed across regions.

**Table 1:** Selected characteristics of women with breast cancer in the aggregate cohort, and from each participant region. Italy, 2011–2016.

	Aggregate cohort (N = 16,753)	Lombardy (N = 8944)	Emilia-Romagna (N = 4323)	Lazio (N = 3486)	p-value^b^
Age					
18–49	3764 (22.5%)	1911 (21.4%)	976 (22.6%)	877 (25.2%)	<0.001
50–64	5412 (32.3%)	2936 (32.8%)	1332 (30.8%)	1144 (32.8%)	
≥65	7577 (45.2%)	4097 (45.8%)	2015 (46.6%)	1465 (42.0%)	
Type of breast cancer					
Invasive cancer	15,407 (92.0%)	8223 (91.9%)	3878 (89.7%)	3306 (94.8%)	<0.001
Carcinoma in situ	1346 (8.0%)	721 (8.1%)	445 (10.3%)	180 (5.2%)	
Type of surgery					
Breast-conserving surgery	12,229 (73.0%)	6352 (71.0%)	3304 (76.4%)	2573 (73.8%)	<0.001
Mastectomy	4524 (27.0%)	2592 (29.0%)	1019 (23.6%)	913 (26.2%)	
Multisource Comorbidity Score^a^					
0–2	10,471 (62.5%)	6159 (68.9%)	2631 (60.9%)	1681 (48.2%)	<0.001
3–5	4867 (29.1%)	2221 (24.8%)	1309 (30.3%)	1337 (38.3%)	
6–8	1044 (6.2%)	412 (4.6%)	274 (6.3%)	358 (10.3%)	
9–11	234 (1.4%)	93 (1.0%)	68 (1.6%)	73 (2.1%)	
≥12	137 (0.8%)	59 (0.7%)	41 (0.9%)	37 (1.1%)	

a According to drugs dispensed and hospital admissions within 3 years from index hospitalization.

b According to the chi-square test.

### 3.2 Adherence with recommendations

Adherence with recommendations of cohort members from each participant region, as well as of the aggregate cohort, is shown in Table 2. The most closely followed recommendation was appropriateness of complementary radiotherapy (82%), followed by surgery timeliness (74%), appropriateness of mammographic follow-up (73%) and finally medical therapy timeliness (53%). Cohort members from Lazio on average exhibited lower adherence than those from Lombardy and Emilia-Romagna.

**Table 2:** Adherence with selected recommendations among women who underwent surgery for breast cancer. Italy, 2011–2016.

	Lombardy	Emilia-Romagna	Lazio	Overall
Timeliness of surgery a	5253 (75.0%)	2457 (72.3%)	968 (69.0%)	8678 (73.5%)
Timeliness of medical therapy b	3737 (53.9%)	1944 (63.3%)	1135 (41.1%)	6816 (53.4%)
Complementary radiotherapy c	4102 (84.0%)	2137 (85.2%)	1531 (72.8%)	7770 (81.8%)
Follow-up mammogram d	7082 (81.9%)	2933 (69.9%)	1794 (53.3%)	11,809 (72.8%)

a Cohort members who in the six months before index hospitalization underwent mammography and did not receive neoadjuvant therapy, were classified as adherent with surgery timeliness if they underwent mammography at least once in the 2 months before index hospitalization, otherwise they were classified as non-adherent (7006 in Lombardy, 3400 in Emilia-Romagna and 1403 in Lazio).

b Cohort members who did not receive neoadjuvant therapy in the six months before index hospitalization, did not receive exclusive radiotherapy within six months after index discharge, were not re-hospitalized for breast surgery within four months after the index discharge and accumulated at least 45 days of follow-up, were classified as adherent with the medical therapy timeliness recommendation if they started chemotherapy within 45 days after index discharge, otherwise they were classified as non-adherent (6928 in Lombardy, 3072 in Emilia-Romagna and 2763 in Lazio).

c Cohort members who had a diagnosis of invasive breast cancer, underwent breast-conserving surgery during the index hospital stay, used chemotherapy within six months after index discharge, and accumulated at least twelve months of follow-up, were classified as adherent with recommendation of appropriate complementary radiotherapy if they started radiotherapy within twelve months from the index discharge, otherwise they were classified as non-adherent (4883 in Lombardy, 2508 in Emilia-Romagna and 2102 in Lazio).

d Cohort members who accumulated at least eighteen months of follow-up, were classified as adherent with the appropriate mammographic follow-up recommendation if they underwent at least one mammography within eighteen months from index discharge, otherwise they were classified as non-adherent (8652 in Lombardy, 4196 in Emilia-Romagna and 3367 in Lazio).

### 3.3 Association between adherence and mortality

**Fig. 2:**
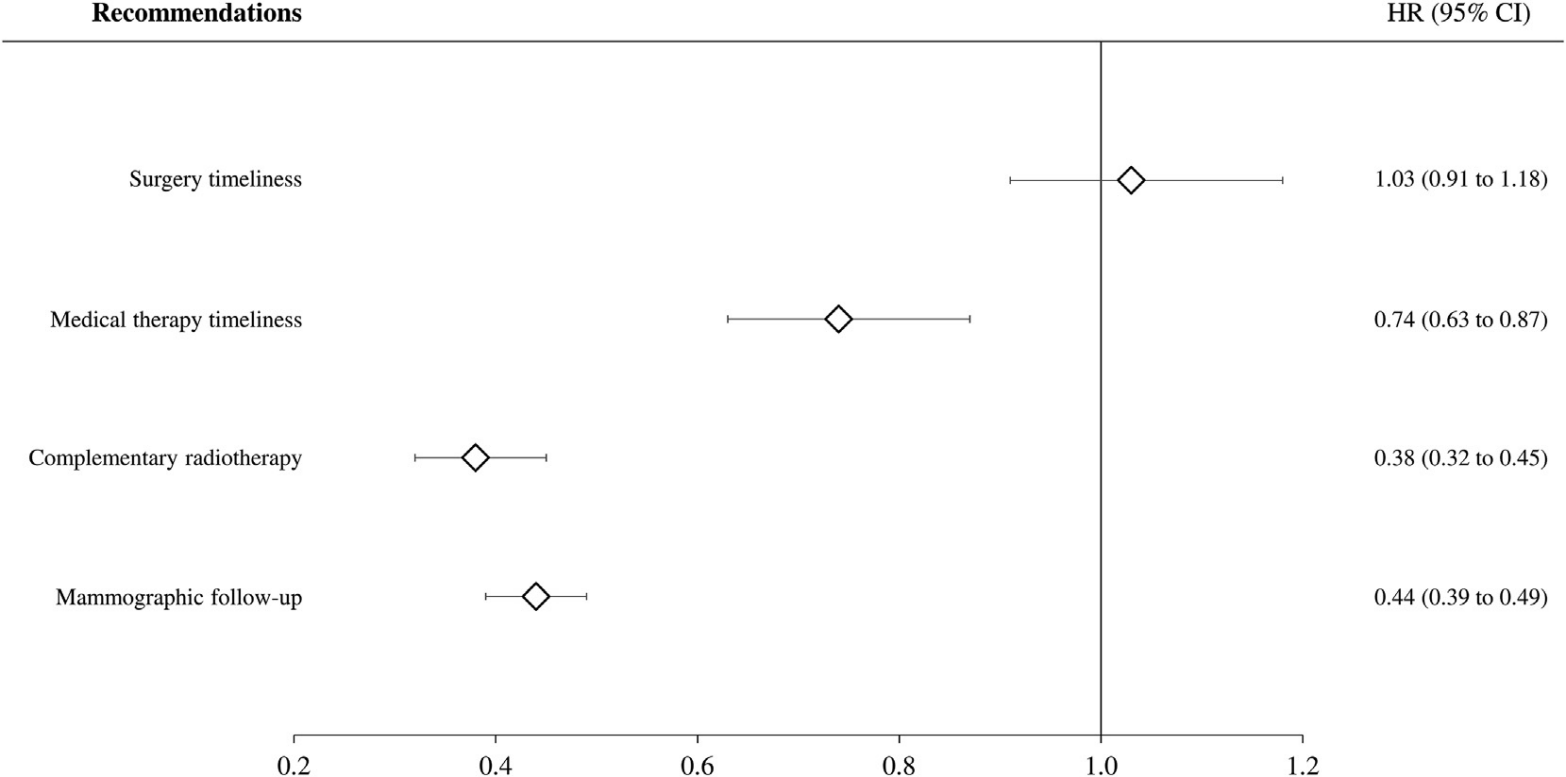
Forest plots of summarized hazard ratios (HR) for the association between adherence with selected recommendations and all-cause mortality. Italy, 2011–2016. Footnote. See footnote to Table 2 for adherence definitions. Region-specific HR, and 95% confidence intervals, were estimated by fitting a Cox proportional hazard model, adjusting for covariates listed in Table 1. The random-effects model was used to obtain the summarized estimates.

**Fig. S1:**
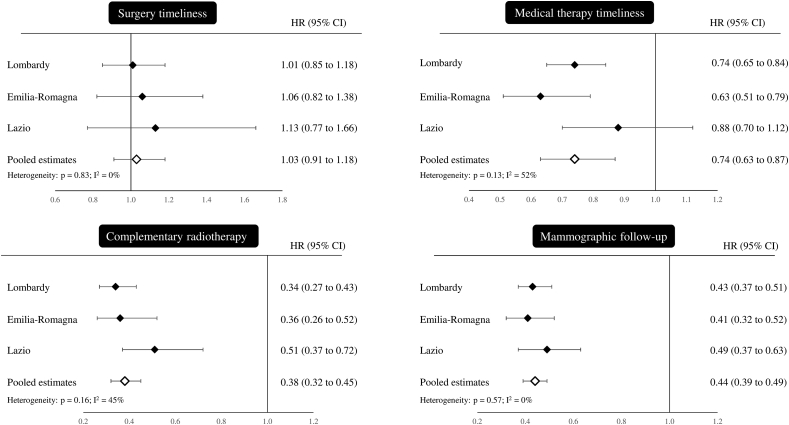
Forest plots of region-specific (black diamonds) and summarized (white diamonds) hazard ratios (HR) for the association between adherence with selected recommendations and all-cause mortality. Italy, 2011–2016. Footnote. See footnote to Table 2 for adherence definitions. HR, and 95% confidence intervals (represented by horizontal lines), were estimated by fitting a Cox proportional hazard model. Estimates were adjusted for covariates listed in Table 1. A random effects model was used for summarized estimates. P-values and I^2^ testing and measuring for heterogeneity between region estimates are reported

Forrest plots for the adherence-outcome relationships are shown in Fig. 2. Adherence with medical therapy timeliness and appropriateness of both complementary radiotherapy and mammographic follow-up exerted protective effects on mortality with risk reductions of 26% (95% CI, 13%–37%), 62% (55%–68%) and 56% (51%–61%), respectively. Conversely, there was no evidence that mortality was affected by surgery timeliness. No evidence of between-region heterogeneity was observed, with I^2^ values ranging from 0% to 52% (Supplementary Fig. S1).

**Fig. 3:**
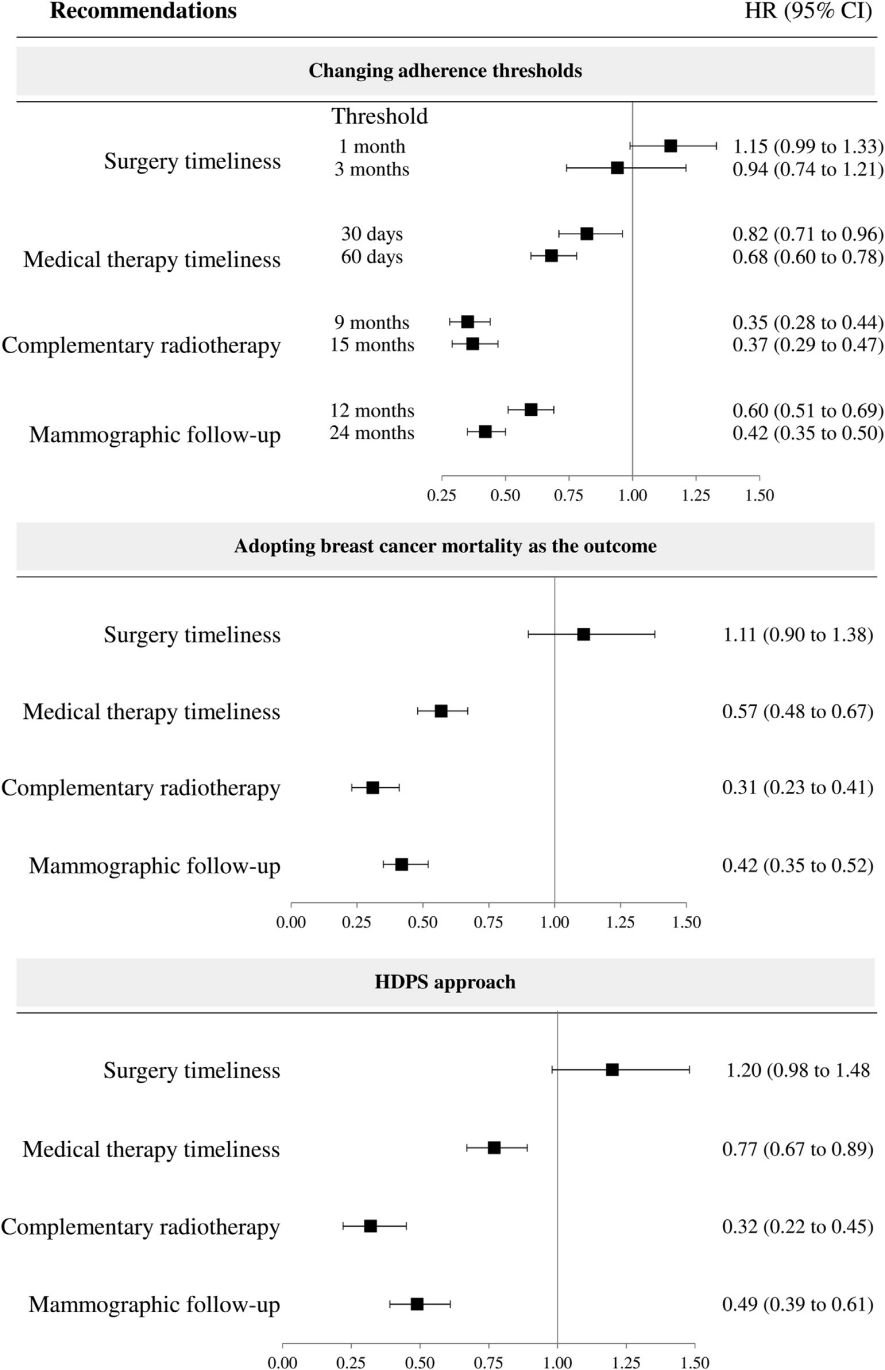
Influences of modifying criteria adherence and outcome definition, and adopting a High Dimensional Propensity Score (HDPS) matching design on the observed hazard ratios (HR), and 95% confidence interval (CI) for adherence with selected recommendations associated with all-cause mortality. Italy, 2011–2016. Footnote. See footnote to Table 2 for definitions of adherence used in the main analysis. Modified criteria related to (i) surgery timeliness: undergoing mammography at least once in the 1 or 3 months before index hospitalization, rather than 2 months as in the main analysis, (ii) medical therapy timeliness: starting chemotherapy within 30 or 60 days after index discharge, rather than 45 days as in the main analysis, (iii) appropriateness of complementary radiotherapy: starting radiotherapy within 9 or 15 months after index discharge, rather than 45 days as in the main analysis, and (iv) appropriateness of mammographic follow-up within 12 or 24 months after index hospitalization, rather than 18 months as in the main analysis. See text for details on High Dimensional Propensity Score (HDPS) matching design.

HRs obtained from the main analyses did not change substantially by (i) modifying criteria for defining adherence, (ii) limiting the outcome to breast cancer mortality only, nor by (iii) adopting an HDPS matching design (Fig. 3).

## 4 Discussion

This study confirms previously reported findings, as well as highlighting new ones. As far as confirmation of previous findings is concerned, we found considerable heterogeneity in breast cancer-related follow-up care across regions. This is consistent with investigations in other developed countries [17], which refer however to private, or mixed public-private, healthcare systems. Although the Italian NHS provides universal coverage for many areas of healthcare, including breast cancer, it is likely that regional disparities reflect differences in the quality of care provided by public services, indicating that robust intervention is required to ensure good clinical support for NHS beneficiaries throughout the country.

As far as new findings are concerned, our study shows that almost all the adopted measurable indicators of quality of care for women operated for breast cancer are associated with improved overall survival. The importance of associations was not trivial because, compared with women who did not adhere with recommendations, those who adhered presented reduced mortality of 26% (medical therapy timeliness), 56% (appropriateness of mammographic follow-up) and 62% (appropriateness of complementary radiotherapy), suggesting that the investigated factors may, at least partly, explain differences in survival across European countries [18], as well as across Italian Regions [19]. We did not find that overall survival declines when surgery timeliness improves, as found in other cancer studies [20]. However, it has been documented that in breast cancer patients the negative association between time to surgery and survival affects women in stage I and II but not those in stage III, probably because baseline mortality of the latter is too great for an understanding of the effect imposed by a delay in treatment [21]. Considering that HCU data do not allow women to be stratified according to breast cancer stage, our findings suggest that surgery timeliness is not suitable for inter-region comparisons. Of course, this does not mean that efforts to minimize preoperative delay for all patients are not advisable.

Our study was developed with the backing of the Italian Health Ministry with the aim of obtaining a simple tool to understand regional variations in the management of women operated for breast cancer. It required the availability of good quality data in order to (i) capture women newly taken in care; (ii) characterize them as far as possible in terms of demographic and clinical features; (iii) outline their use of recommended clinical services; and (iv) identify those who experience relevant clinical outcomes. This was made possible because in Italy an automated system of databases providing information on essential healthcare is available in each of the 21 administrative units (19 regions and 2 autonomous provinces) for the management of the publicly funded healthcare system serving virtually all citizens. Because of constraints limiting the free movement of electronic health data even within the same country [22], a two-stage procedure allowing for regional data processing and subsequent pooling of aggregate data, was adopted. Assuming comparability in data quality, estimate accuracy is provided by the procedure [12].

Our findings are based on HCU data covering NHS beneficiaries in several jurisdictions with the same healthcare framework, but different approaches to healthcare delivery. HCU data can provide more accurate estimates of medical care than alternative approaches based on service-based data. Comparative analysis of these data permits direct assessment of the relative performance across regions in the delivery of guideline-based care to the population [23]. Although the results are generated by the Italian health system, they provide real-world data in a total geographically-defined adult breast cancer population.

On the other hand, the main limitation of the study is the paucity of data on individual characteristics and clinical features. Since adherent women are expected to be different from those who did not adhere to recommendations in several ways, our results could be affected by confounding. That is, the all-cause mortality reduction associated with closer adherence with recommendations might have been generated by uncontrolled factors, accompanying but different from closer adherence. For example, radiotherapy, as well as mammographic checks, might have been followed mainly by patients suffering from more severe breast cancer. However, as the latter are at a higher baseline risk of experiencing the outcome, the protective action of regular checks is expected to be higher than that observed in our study. In addition, our main findings did not change substantially with a HDPS matching design. Of course, this does not entirely eliminate the problem of confounding, one aspect of which is that adherence may be a surrogate for overall health-seeking behaviour. In addition, it has been shown that socio-economic status affects accessibility to population-based screening programs, even in a country with universal coverage for many areas of healthcare [24], including breast cancer screening [25]. Overall, available evidence suggest patients who adhere more closely to both healthcare and screening programs might have followed healthy lifestyle advice more regularly and been more effectively treated. If this were true, however, we should conclude that the considered indicators are a surrogate of the quality of care provided to women operated for breast cancer, which is exactly what is requested of them. The observation that each indicator is associated with survival approximately with the same intensity in the considered regions probably means that their ability to assess the quality of care is uniform across regions.

## 5 Conclusions

In summary, because benefits are expected from improving adherence with the recommendations considered, close control of women operated for breast cancer through medical care timeliness and appropriateness of radiotherapy and mammographic monitoring must be considered the cornerstone of national guidance, national audits, and quality improvement incentive schemes.

## Funding

This study was funded by a research grant from the Italian Health Ministry through two independent sources: (i) “Modelli per il monitoraggio e la valutazione delle cure integrate (CI) nell’ambito del Nuovo Sistema di Garanzia dell’assistenza sanitaria“ project (grant number J59H06000160001), (ii) ‘Ricerca Finalizzata 2016’ (NET- 2016–02363853). Data analyses were performed at the Laboratory of Healthcare Research & Pharmacoepidemiology, 10.13039/501100002954University of Milano-Bicocca with grants from the Italian 10.13039/501100003407Ministry of Education, University and Research (‘Fondo d’Ateneo per la Ricerca’ portion, year 2018).

The funding source had no role in the design of the study, the collection, analysis and interpretation of the data, or the decision to approve publication of the finished manuscript.

## Ethics approval

Under the rules of the Italian Drugs Agency (available at: http://www.agenziafarmaco.gov.it/sites/default/files/det_20marzo2008.pdf), retrospective studies using administrative databases do not require Ethics Committee protocol approval.

## Declaration of competing interest

Giovanni Corrao received research support from the European Community (10.13039/501100000780EC), the Italian Drugs Agency (10.13039/501100003197AIFA), and the Italian 10.13039/501100003407Ministry of Education, University and Research (MIUR). He took part in a number of projects funded by pharmaceutical companies (i.e., 10.13039/100004336Novartis, GSK, 10.13039/100004337Roche, AMGEN and BMS). He also received honoraria from Roche as a member of the Advisory Board.

For the remaining authors, nothing was declared.
