# Supplementary material for: Influence of adherence with guideline-driven recommendations on survival in women operated for breast cancer: Real-life evidence from Italy
Source: Breast. 2020 Jul 1;53:51–8. doi: 10.1016/j.breast.2020.06.010 (PMC7375570; doi:10.1016/j.breast.2020.06.010)
Supplement: Multimedia component 1 [file mmc1.docx]

**Supplementary Table S1.** Diagnoses and drugs codes used for the study purpose

|  | Diagnoses | ICD-CM codes |
| --- | --- | --- |
| *Case identification ^†^* | | |
| Invasive cancer | | 174.* |
| Carcinoma in situ | | 233.0 |
| Breast-conserving surgery | | 85.20-85.25 (procedure codes) |
| Mastectomy | | 85.33-85.36, 85.4* (procedure codes) |
| *Exclusion criteria ^†^* | | |
| Previous breast cancer | | V10.3, 174*, 233.0 |
| Other cancer | | 140*-172*, 176*-195, 200-208*, V.10* (except V10.3) |
| Malignant neoplasm | | 197.*-199.* |
| *Recommendations ^†^* | | |
| Chemotherapy | | V58.1  99.25, 99.28 (procedure codes) |
| Mammography | | 87.37 (procedure codes) |
| Radiotherapy | | V58.0  92.2* (procedure codes) |
| *Cause of death ^‡^* | | |
| Breast cancer | | C50 |
| Drugs | | **ATC codes** |
| Antineoplastic agents | | L01 |
| Endocrine therapy | | L02 |
| Outpatient services | | **National codes** |
| Chemotherapy | | 99.24.1, 99.25 |
| Mammography | | 87.37.1, 87.37.2 |
| Radiotherapy | | 92.2*, 92.47.8, 92.47.9 |

† According to the ICD-9-CM (International Classification of Disease, 9th Revision) system

‡ According to the ICD-10-CM (International Classification of Disease, 10th Revision) system

**Supplementary Table S2.** Weights of conditions contributing to the Multisource Comorbidity Score (for cancer patients)

| Disease/condition | Weight |
| --- | --- |
| Tuberculosis | 9 |
| Drug addition | 9 |
| Weight loss | 8 |
| Dementia | 6 |
| Liver disease | 6 |
| Disorders of fluid, electrolyte, and acid-base balance | 4 |
| Alcohol abuse | 4 |
| Other neurological diseases | 4 |
| Hemiplegia and hemiparesis | 3 |
| Anxiety medication | 3 |
| Anaemias | 3 |
| Psychoses | 3 |
| Kidney diseases | 3 |
| Cerebrovascular diseases | 2 |
| Kidney dialysis | 2 |
| Diabetes | 2 |
| Heart failure | 2 |
| Chronic pulmonary diseases | 2 |
| Peptic ulcer | 2 |
| Coagulation defects | 1 |
| Vascular diseases | 1 |
| Pain and inflammation | 1 |
| HIV infection | 1 |
| Depression | 1 |
| Kidney transplantation | 1 |

The ICD-9 and ATC codes of these conditions are reported in Corrao G, et al. BMJ Open. 2017;7:e019503. doi: 10.1136/bmjopen-2017-019503
